# Correction to: Unpacking policy formulation and industry influence: the case of the draft control of marketing of alcoholic beverages bill in South Africa

**DOI:** 10.1093/heapol/czae026

**Published:** 2024-04-12

This is a correction to: Adam Bertscher, Leslie London, Marsha Orgill, Unpacking policy formulation and industry influence: the case of the draft control of marketing of alcoholic beverages bill in South Africa, *Health Policy and Planning*, Volume 33, Issue 7, September 2018, Pages 786–800, https://doi.org/10.1093/heapol/czy049

The corresponding author’s contact details have been updated.

